# Bayesian approach for the estimation of cyclosporine area under the curve using limited sampling strategies in pediatric hematopoietic stem cell transplantation

**DOI:** 10.1186/1742-4682-11-39

**Published:** 2014-09-05

**Authors:** Sarem Sarem, Jun Li, Olivier Barriere, Catherine Litalien, Yves Théorêt, Anne-Laure Lapeyraque, Fahima Nekka

**Affiliations:** 1 Faculty of Pharmacy, Université de Montréal, C.P. 6128, Succ. Centre-ville, H3C 3J7 Montreal, Canada; 2 Clinical Pharmacology Unit, CHU Ste-Justine, Montreal, Canada; 3 Centre de Recherches Mathématiques, University Montréal, Montreal, Canada; 4 Centre for Applied Mathematics in Biosciences and Medicine, McGill University, Montreal, Canada; 5 Department of Pediatrics, CHU Ste-Justine, Montreal, Canada; 6 Department of Biochemistry, CHU Ste-Justine, Montreal, Canada; 7 Department of Pharmacology, Université de Montréal, Montreal, Canada

**Keywords:** Bayesian approach, Population pharmacokinetics (Pop-PK), Cyclosporine (CsA), Area under the curve (AUC), Limited sampling strategy (LSS)

## Abstract

**Background:**

The optimal marker for cyclosporine (CsA) monitoring in transplantation patients remains controversial. However, there is a growing interest in the use of the area under the concentration-time curve (AUC), particularly for cyclosporine dose adjustment in pediatric hematopoietic stem cell transplantation. In this paper, we develop Bayesian limited sampling strategies (B-LSS) for cyclosporine AUC estimation using population pharmacokinetic (Pop-PK) models and investigate related issues, with the aim to improve B-LSS prediction performance.

**Methods:**

Twenty five pediatric hematopoietic stem cell transplantation patients receiving intravenous and oral cyclosporine were investigated. Pop-PK analyses were carried out and the predictive performance of B-LSS was evaluated using the final Pop-PK model and several related ones. The performance of B-LSS when targeting different versions of AUC was also discussed.

**Results:**

A two-compartment structure model with a lag time and a combined additive and proportional error is retained. The final covariate model does not improve the B-LSS prediction performance. The best performing models for intravenous and oral cyclosporine are the structure ones with combined and additive error, respectively. Twelve B-LSS, consisting of 4 or less sampling points obtained within 4 hours post-dose, predict AUC with 95^th^ percentile of the absolute values of relative prediction errors of 20% or less. Moreover, B-LSS perform better for the prediction of the ‘underlying’ AUC derived from the Pop-PK model estimated concentrations that exclude the residual errors, in comparison to their prediction of the observed AUC directly calculated using measured concentrations.

**Conclusions:**

B-LSS can adequately estimate cyclosporine AUC. However, B-LSS performance is not perfectly in line with the standard Pop-PK model selection criteria; hence the final model might not be ideal for AUC prediction purpose. Therefore, for B-LSS application, Pop-PK model diagnostic criteria should additionally account for AUC prediction errors.

## Background

Therapeutic drug monitoring is a common practice for the use of immunosuppressant drugs, which generally exhibit considerable inter- or intra- pharmacokinetic (PK) variability and narrow therapeutic window [1]. A non-monitored dosing can increase the risk for therapeutic failure or induce serious undesirable effects. Currently, therapeutic drug monitoring approach, which involves the measurement of drug concentrations and their interpretation, has become a standard of care in immunosuppressant therapy for dose optimization, with the aim of maximizing therapeutic benefits and minimizing adverse effects [1,2]. In clinical practice, the pre-dose concentration (C_0_) is widely used as a PK marker for the therapeutic drug monitoring due to its accessibility. Nonetheless, treatment failure, adverse effects, and toxicity can still arise even in situations where C_0_ is within the recognized therapeutic range [3,4]. These risks call for the implication of other PK based surrogates, such as the area under the concentration-time curve (AUC) which is generally known as the best indicator of drug systemic exposure. While its use as an optimal marker for immunosuppressant agents monitoring remains controversial its correlation with clinical outcomes is increasingly being investigated [5-7].

When estimating AUC, we generally refer to the observed AUC, usually denoted AUC_obs,_ which is obtained using the trapezoidal method. This method can be cumbersome for patients and their care providers since it requires a frequent sampling over a time interval long enough to fully represent the drug disposition. As an alternative, limited sampling strategies (LSS) have been proposed to predict AUC with an adequate precision, using a reduced number of sampling points drawn within a short time interval. LSS have been applied with two main approaches, namely, the multiple linear regression-based LSS (R-LSS) and Bayesian-based LSS (B-LSS).

The regression approach aims to establish a linear relationship between one or more concentration-time points (independent variables) and AUC (dependent variable) in the form of the following equation:

$$\mathrm{AU}C_{\text{pred}}=F_{0}+F_{1}\times C_{t_{1}}+\ldots +F_{k}\times C_{t_{k}}$$

where C_t1_, C_t2_, …, C_tk_ are the concentrations sampled at times t_1_, t_2_, …, t_k_, respectively; and F_0_, F_1_, …, F_k_ are regression coefficients. For its simplicity, the use of regression LSS is widely spread as a bedside application. However, its use is highly restrictive since samples are assumed to be taken on nominal sampling times, excluding thus any possible deviation.

**Figure 1:**
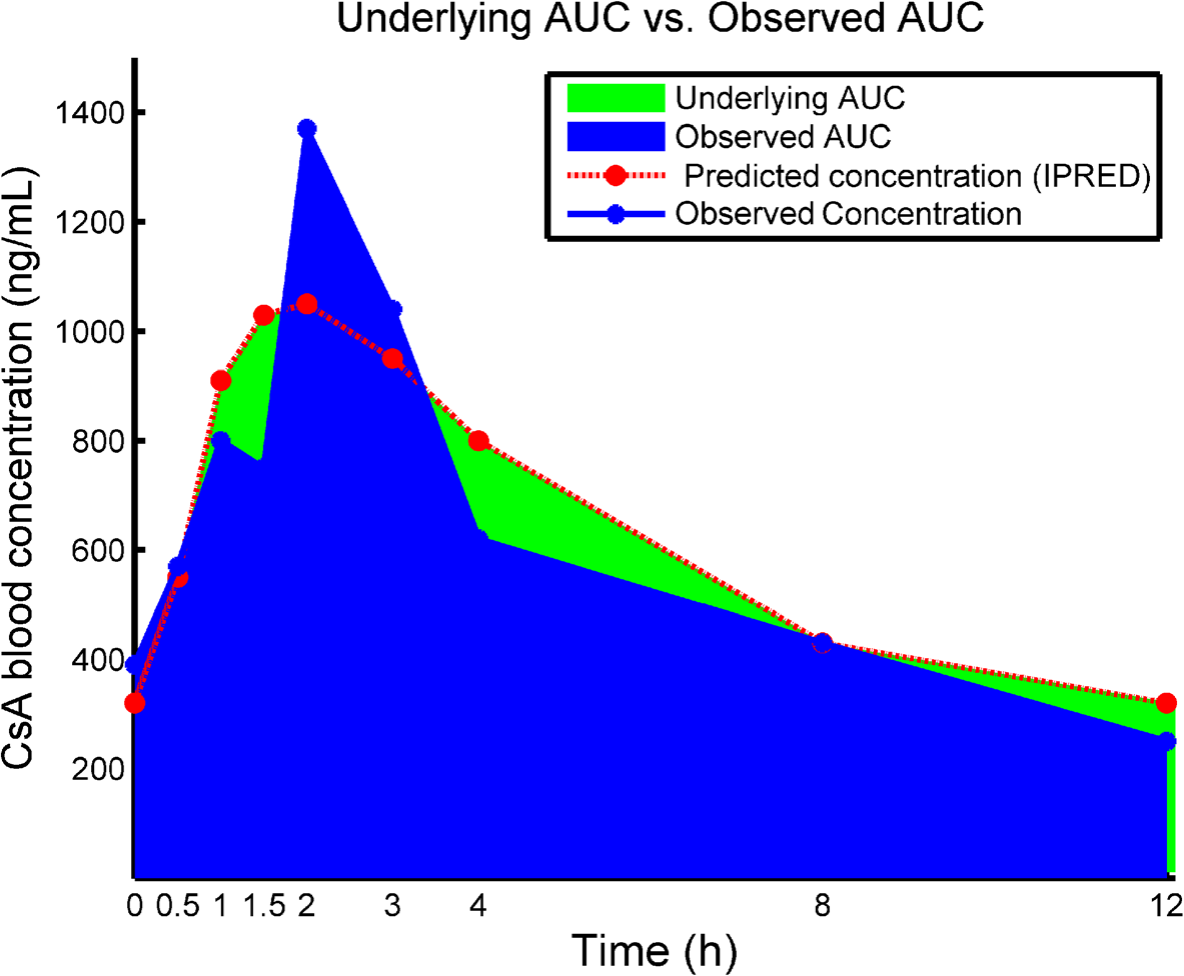
Underlying AUC (7448 ng.h/ml) Vs. Observed AUC (7017 ng.h/ml).

The B-LSS approach requires the use of several drug concentrations in addition to a well-established population pharmacokinetic (Pop-PK) model for the estimation of AUC. This model, considered as the acquired *prior* knowledge of drug characteristics, helps to improve the estimation, otherwise solely based on the observed drug concentrations. With the B-LSS method, the estimated individual PK parameters are obtained using the empirical Bayesian approach; these parameters are then used for the prediction of drug concentrations and, consequently, the estimation of AUC. One advantage of the Bayesian approach over the regression LSS is its flexibility in terms of sampling time deviations which are readily considered when building the associated Pop-PK model and predicting the individual PK parameters; since the real sampling times can be used in case of sampling deviations from the nominal times. Nevertheless, the use of B-LSS can be hampered by the need for trained professionals and specialized software. This situation is however changing progressively since many PK software packages with user-friendly interfaces are now made available.In both LSS approaches, the estimation of AUC aims to approximate the real area under the curve that could be reachable in ideal conditions of frequent blood samplings associated with perfect measurements that reflect precisely drug concentrations. However, only few samples are usually available. In addition, these samples are generally affected by different sources of errors, emanating from sample collection, measurement method, and data processing. These limitations can potentially be inherited by the observed AUC, and consequently raising the question of its reliability. It would be thus interesting to alternatively consider the AUC calculated directly from the estimated individual concentrations using the Pop-PK model, assuming the exclusion of the residual errors. These estimated concentrations are denoted IPRED in the usual notation of NONMEM^®^, the mostly used software in Pop-PK analyses. We refer to this AUC as ‘underlying’ AUC. The difference between observed AUC and ‘underlying’ AUC is illustrated in Figure 1. Although ‘underlying’ AUC cannot be directly measured in practice, we believe that it represents the intrinsic property of a patient’s PK profile as it is not altered by residual errors and hence can be a better predictor for drug effects, compared to the observed AUC where the residual errors are always present.

Cyclosporine (CsA) is a typical example of immunosuppressive agents where LSS are widely used. Therapeutic drug monitoring is recommended for CsA dose adjustment because of its large PK variability and small therapeutic index [2,8]. CsA is used mainly in hematopoietic stem cell transplantation for the prophylaxis of graft-versus-host disease. In this context, there is a growing interest in the use of AUC as a therapeutic drug monitoring marker [6,7]. However, prospective trials are still needed to evaluate the efficacy of AUC guided dose adjustment.

In hematopoietic stem cell transplantation, graft-versus-host disease can result in diffuse inflammation that affects intestinal integrity, thus causing reduction and delay in CsA absorption, while the clearance is reported higher in comparison to solid organ transplantation [9,10]. Recently, LSS have been applied by several research groups to predict AUC for hematopoietic stem cell transplantation in adults [11-14]. However, their results cannot be directly transferred to pediatric patients who generally require higher doses, as they have faster systemic clearance and lower CsA exposure [9,10,15,16]. Therefore, particular B-LSS in pediatric patients need to be developed and validated.

To our knowledge, only three LSS studies for the prediction of CsA AUC in pediatric hematopoietic stem cell transplantation have been published. Willemze et al. found a good performance using B-LSS for intravenous (IV) and oral (PO) CsA; however, they were tested on a small number of combinations of sampling points, with no validation reported [17]. Based on a population of 24 pediatric patients receiving 2 hours BID infusion, Sibbald et al. reported regression LSS [18]. These LSS are developed only for PK profiles obtained after the first CsA IV dose and their application is restricted to this particular condition. Recently, Dupuis et al. validated these LSS and reported new regression LSS for the prediction of AUC at the steady state [19]. The latter LSS showed a good performance but required samples to be drawn within 8 hours post-dose and were developed and validated only for IV CsA.

In this paper, we will develop practical B-LSS for the prediction of AUC in pediatric hematopoietic stem cell transplantation patients after IV and PO CsA administrations. In this context and based on available PK data of our pediatric population, we developed Pop-PK models of CsA following the general Pop-PK modeling steps, but with a particular care for its intended use in AUC prediction by B-LSS. Furthermore, to insure the LSS applicability in clinical settings, the number of concentration-time points and sampling duration were restricted to 4 points or less drawn within 4 hours post-dose. Performance of these LSS is evaluated using well established error indices.

## Materials and methods

### Patients

Pediatric patients receiving IV (2 hours infusion) or PO CsA twice daily for graft-versus-host disease prophylaxis after undergoing hematopoietic stem cell transplantation from a sibling or unrelated donor, at the Centre Hospitalier Universitaire Sainte-Justine, were considered for inclusion in this retrospective study. Patients who were 19 years old or more were excluded. The study was approved by the institutional research ethics committee at the Centre Hospitalier Universitaire Sainte-Justine. Twenty five pediatric patients were eligible for inclusion in this study over a period from August 2009 to August 2010. Eighteen of these patients have IV and PO pharmacokinetic profiles. Patients’ characteristics are summarized in Table 1.

**Table 1:** Patients’ information summary

**Parameter (unit)**	**Number or median (range)**	
	**IV**	**PO**
Patients	19	20
Sex: male/female	10/9	12/8
Age at transplantation (year)	10.5 (1–18)	11.1 (0.5–18.2)
Transplantation type: Sibling/Unrelated	10/9	13/7
Included PK profiles	23	39
Formulation	23 (IV)	19 (Susp)/20 (Cap)
Time post transplantation (month)	0.13 (0.1–1.7)	1.28 (0.7–9.1)
Age at PK profile (year)	10.4 (1–17.9)	11.9 (1.2–18.3)
Weight (kg)	33 (10–81)	38 (8–83)
Cyclosporine dose (mg/kg/day)	2.5 (1–3.2)	4.2 (1–8.3)
Concomitant corticosteroid	13	26^†^
Albumin (g/L)	32 (19–48)	32 (22–41)^‡^
Creatinine (mmol/L)	33 (12–358)	50 (13–117)^‡^
Bilirubin (μmol/L)	11 (5–64)	10 (3–596)^‡^
AST (U/L)	20 (9–42)	24 (13–125)^‡^
ALT (U/L)	24 (9–85)	31 (19–69)^‡^
GGT (U/L)	35 (8–94)	32 (9–217)^‡^
AP (U/L)	87 (1.9–203)	110 (53–302)^‡^
Hb (g/dL)	93 (64–143)	87 (64–122)^‡^
Hct (%)	25 (18–44)	26 (19–36)^‡^

ALT: alanine aminotransferase; AP: alkaline phosphatase; AST: aspartate aminotransferase; Hb: hemoglobin; Hct: hematocrit; IV: intravenous administration; GGT: γ- glutamyltranspeptidase; PK: pharmacokinetic; PO: oral administration; Susp: suspension; Cap: capsule.

^†^Data available for 37 profiles.

^‡^Data available for 38 profiles.

### Cyclosporine dose adjustment

Since 2010, the medical team at the Centre Hospitalier Universitaire Sainte-Justine caring for hematopoietic stem cell transplantation patients moved from C_0_- to AUC-based monitoring in light of controversy regarding the usefulness of dose adjustments based on CsA C_0_[20]. Hence, CsA dose adjustments were made by the treating physician in accordance with institutional target AUC_0-12h_ values, which were defined based on published data from renal transplantation studies [21,22] and one adult hematopoietic stem cell transplantation study [23]. These were adapted by the team according to the patient’s underlying diseases.

### PK data

**Figure 2:**
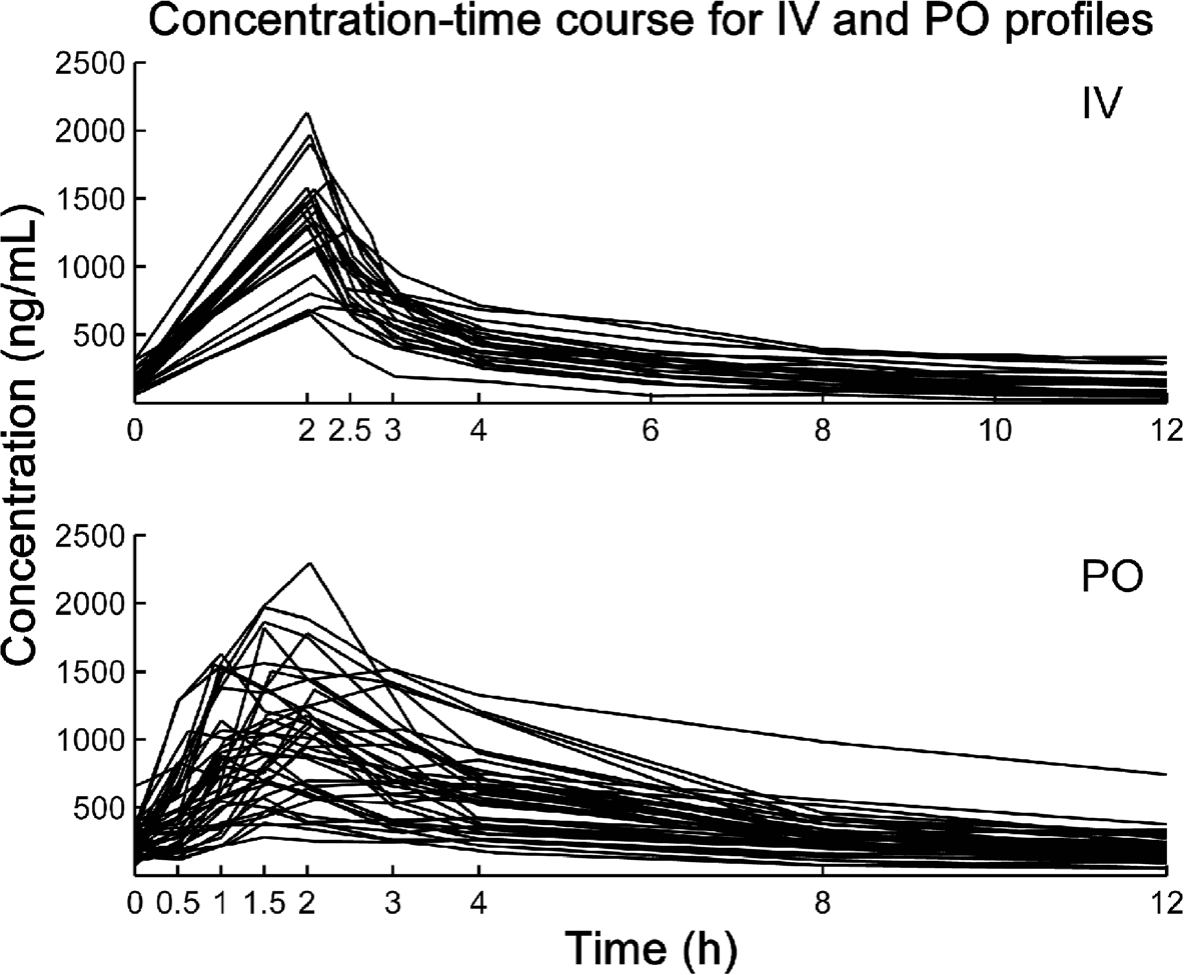
Concentration-time courses for the available full profiles.

All available steady state PK profiles that contained at least 7 concentration-time points were incorporated in this study for a total of 23 IV and 39 PO profiles. Blood samples were drawn before and at 2, 3, 4, 6, 8, 10, and 12 hours after CsA administration for IV profiles and at 0.5, 1, 1.5, 2, 3, 4, 8 and 12 hours after CsA administration for PO profiles. Concentrations were measured using ARCHITECTi2000SR^®^ (Abbott Laboratories, Abbott Park, Illinois, USA). The lower and upper limits of detection were 30 and 1500 ng/mL, respectively. The between-run coefficients of variation were 9.95% at 87 ng/mL, 8.64% at 340 ng/mL, and 9.25% at 850 ng/mL. Blood samples with CsA concentrations > 1500 ng/mL were diluted with blank blood. The associated observed AUC were calculated using the trapezoidal method. Individual CsA concentration-time profiles are reported in Figure 2.

### Development of Pop-PK model

Population PK analyses were performed using the nonlinear mixed effect approach as implemented in NONMEM^®^ software (Version VII). The first order conditional estimation with interaction (FOCE-I) method was used to determine PK parameters and the associated variability. To define the structural model, one, two and three compartment models with first-order absorption and elimination were used to analyze available CsA data. The lag time in absorption was also tested for each model. The exponential model was used to describe inter-individual variability for PK parameters as expressed in Eq. 1:

(1) $$\theta_{\mathrm{ij}}=\theta_{j}\times \;\text{EXP}\left(\eta_{\mathrm{ij}}\right)$$

where θ_ij_ is the j^th^ PK parameter for the i^th^ individual, θ_j_ is the typical value of the population parameter; ɳ_ij_ is a random variable characterizing the between subject variability. A combined version of additive and proportional models was used to test for residual variability (Eq. 2):

(2) $$C_{\text{obs}}=\;C_{\text{pred}}\times \;\left(1+\epsilon_{1}\right)\;+\epsilon_{2}$$

where C_obs_ and C_pred_ are the observed and predicted CsA blood concentrations, respectively; ε_1_ and ε_2_ are random variables describing the unexplained residual variability.

The structural model was developed based on statistical significance in the reduction of the objective function value (OFV) using likelihood ratio (LT) test, as well as other standard indicators such as the model stability and the improvement in model fitting. As usually done in pediatric Pop-PK modeling, weight had been initially integrated as an allometric scaling factor for the clearance and the volume of distribution [24]. The covariate model was then established by the forward inclusion backward elimination strategy, using Perl speaks NONMEM (PsN) script [25], in which a change of OFV greater than 6.63 and 7.87, associated with a p-value of 0.01 and 0.005, was used as selection criteria for statistical significance, respectively. A total number of 19 covariates were included in the plan. With a careful checking of graphical relationship and consideration of their clinical meaning, potentially meaningful covariates were tested (see model development details in Appendix).

### B-LSS development and validation

Using the nine available sampling points of each PK profile included in this study, we evaluated the performance of all possible combinations that contain one, two, three, or four concentration-time points, which gives rise to a total number of 255 LSS to be tested. These LSS were divided into four subgroups according to the number of concentration-time points included in the LSS plan. To allow validation despite the small number of available data, we used the leave-one-out-cross-validation approach [26].

We briefly recall that, when using the leave-one-out-cross-validation approach, each PK profile is left out in turn from the analysis, which gives rise to a partial dataset noted as Y^(-i)^, i = 1, …, N, where i stands for the temporary excluded i^th^ PK profile. Using the available Pop-PK model of CsA, we estimate the PK parameters associated with the partial data set Y^(-i)^. Then to estimate PK parameters of the excluded profile, the standard empirical Bayesian approach, as implemented in NONMEM^®^, is performed using, as initial values, the Pop-PK parameters previously obtained for Y^(-i)^. This estimation involves the LSS associated concentrations of the excluded profile. These PK parameters obtained for the i^th^ profile are then used to predict its full concentration-time course that includes the 9 concentration-time points of the sampling protocol. Finally, the predicted AUC for the i^th^ profile is calculated using the trapezoidal method.

Performance of the 255 LSS is evaluated using error indices [27]. For each LSS, relative error (E%), the 95^th^ percentile of the absolute values of relative prediction errors (95^th^ PAE%), mean relative prediction error (ME%) and root mean squared relative prediction error (RMSE%) were calculated. These estimates were based on the following formulations:

(3) $$E_{i}\%=\frac{\mathrm{AU}{C_{\text{pred}}}^{\left(i\right)}-\mathrm{AU}{C_{\text{obs}}}^{\left(i\right)}}{\mathrm{AU}{C_{\text{obs}}}^{\left(i\right)}}\times 100$$

(4) $$\mathrm{ME}\%=\frac{1}{N}\sum_{i}^{N}\frac{\mathrm{AU}{C_{\text{pred}}}^{\left(i\right)}-\mathrm{AU}{C_{\text{obs}}}^{\left(i\right)}}{\mathrm{AU}{C_{\text{obs}}}^{\left(i\right)}}\times 100$$

(5) $$\mathrm{RMSE}\%=\sqrt{\frac{1}{N}{\sum_{i}^{N}\left(\frac{\mathrm{AU}{C_{\text{pred}}}^{\left(i\right)}-\mathrm{AU}{C_{\text{obs}}}^{\left(i\right)}}{\mathrm{AU}{C_{\text{obs}}}^{\left(i\right)}}\right)}^{2}}\times 100$$

95^th^ PAE% = 95^th^ percentile of the increasingly order set:

(6) $${\left\{\left|E_{i}\right|\%\right\}}_{i}^{N}$$

Moreover, since relative errors can induce bias when applied to highly asymmetrical data, the symmetry of the distribution and the range of estimated relative errors were also verified [28].

**Figure 3:**
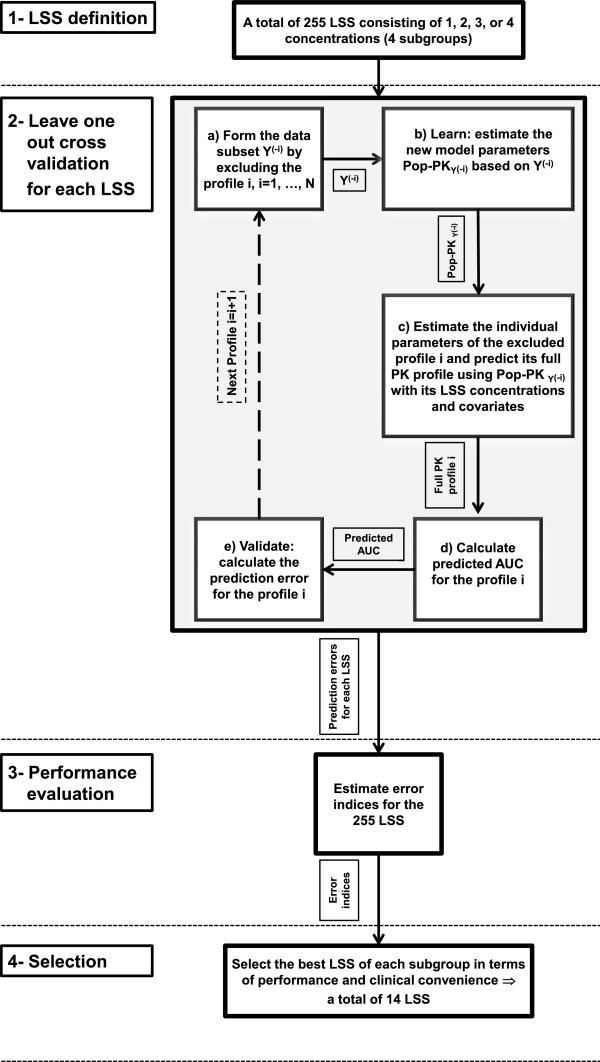
**B-LSS development procedure; Y is the group of all profiles; Y**^**(-i) **^**is the subgroup of all profiles except the i**^**th **^**one, where i = 1,2…,N.**

For each LSS subgroup defined above, four representative B-LSS were chosen to represent the overall performance. In each subgroup, the first chosen B-LSS corresponds to the one that has the highest predictive performance according to 95^th^ PAE%. In addition to this criterion, the following three B-LSS were selected according to two clinically oriented restrictions, namely the inclusion of C_0_ and the limitation of sampling to an interval of 4 hours post-dose. As reported in the Results Section, 28 B-LSS (14 for IV and 14 for PO CsA) are obtained for each evaluated Pop-PK model.Figure 3 depicts the above procedure of B-LSS development and validation.

### Analysis of B-LSS performance

When investigating B-LSS performance using the structural model as well as the final model developed following the standard Pop-PK procedure, we noticed that the final model was not associated with the best performance. This non anticipated result raised the concern about the appropriateness of the final Pop-PK model, with regard to B-LSS application. Indeed, the decision for the final model is mainly determined through objective function value (OFV), a criterion which may not be adequate to optimize a model for B-LSS application. Hence, we decided to investigate the B-LSS performance using intermediate Pop-PK models that differ from the final one in terms of error models and included covariates. To report their B-LSS performance, we chose to use the 95^th^ PAE% for its simplicity and clinical relevance [8]. The results for other performance indices, not reported here for space restriction, were consistent with those of the 95^th^ PAE%.

As mentioned above, we also estimated the ‘underlying’ AUC and used it as a reference for AUC predicted through B-LSS. Then the performance of B-LSS in this context is compared to their performance for the prediction of observed AUC.

The commercial software package MATLAB^®^ (2008b, The Math Works Inc, Natick, Massachusetts, U.S.A.) and NONMEM^®^ (version VII, Icon Development Solutions, Ellicott City, MD) were used for modeling implementation and computations.

## Results

### Final Pop-PK model

The initial model analyses for the description of CsA PK data suggested a two-compartment structure with a combined additive and proportional error model. This structural model was parameterized in terms of: clearance (CL), apparent volume of distribution of the central compartment (Vc), apparent volume of distribution of the peripheral compartment (Vp), inter-compartmental transfer rate (Q), absorption rate (KA), lag time in oral absorption (ALAG), and oral bioavailability (F). Inter-individual variability was estimated for CL, Vc, Q, KA, and F.

Moreover, as usually suggested in pediatric literature, clearance and volume were scaled by weight with powers of ¾ and 1, respectively [24]. With this addition to the structural model, we have performed a standard covariate analysis which led to the final model that included weight (WT), age at profile date (AG), time post transplantation (TPT), alkaline phosphatase (AP), and dosage form (FORM). The details of model construction and estimated parameters can be found in the Appendix.

### Pop-PK model selection based on associated B-LSS performance

The structural model with combined errors (Model 4 in Table 2) and the structural model with additive errors (Model 6 in Table 2) were selected as the best models for performing AUC prediction using B-LSS, for IV and PO profiles, respectively. Their selection was based on their performance in terms of 95^th^ PAE%. Associated to these two models, 16 LSS (11 for IV and 5 for PO) had 95^th^ PAE% of 20% or less.

**Table 2:** Performance of B-LSS for AUC prediction using selected Pop-PK models

	***Model 1***	**95**^**th **^**APE%**	**Model 2**	**95**^**th **^**APE%**	**Model 3**	**95**^**th **^**APE%**	***Model 4***	**95**^**th **^**APE%**	**Model 5**	**95**^**th **^**APE%**	***Model 6***	**95**^**th **^**APE%**
**OFV**	-1900		-1895		-1613		-1790		-1773		-1447	
**IV**	C2, C2.5, C6, C10	*10*	C2, C3, C6, C8	*11*	C2, C2.5, C6, C10	23	C2, C2.5, C8, C10	*7*	C2, C2.5, C8, C10	*8*	C2, C2.5, C4, C6	*15*
	C0, C2, C3, C4	*18*	C0, C2, C3, C4	*18*	C0, C2, C2.5, C4	27	C0, C2, C3, C4	*14*	C0, C2, C3, C4	*14*	C0, C2.5, C3, C4	22
	C0, C2, C2.5, C4	*19*	C0, C2, C2.5, C4	*19*	C0, C2, C3, C4	27	C0, C2, C2.5, C3	*15*	C0, C2, C2.5, C3	*15*	C0, C2, C2.5, C4	24
	C0, C2, C2.5, C3	*19*	C0, C2, C2.5, C3	*19*	C0, C2, C2.5, C3	27	C0, C2, C2.5, C4	*16*	C0, C2, C2.5, C4	*16*	C0, C2, C2.5, C3	24
	C2, C2.5, C10	*12*	C2, C3, C8	*13*	C2, C2.5, C6	24	C2, C2.5, C8	*10*	C2, C2.5, C8	*10*	C2.5, C8, C10	*16*
	C0, C2, C3	*20*	C0, C2.5, C3	*20*	C0, C2, C3	28	C0, C2, C3	*16*	C0, C2, C3	*16*	C0, C2.5, C4	*20*
	C0, C3, C4	*20*	C0, C2, C3	21	C0, C2, C4	28	C0, C2.5, C3	*18*	C0, C2.5, C4	*19*	C0, C2, C4	22
	C0, C2.5, C3	*20*	C0, C3, C4	21	C0, C2.5, C3	28	C0, C2.5, C4	*19*	C0, C2.5, C3	*19*	C0, C2.5, C3	24
	C2.5, C6	*17*	C2.5, C6	*17*	C2.5, C6	27	C2.5, C8	*15*	C2.5, C8	*15*	C2.5, C8	*20*
	C0, C3	21	C0, C3	22	C0, C3	31	C0, C2.5	*20*	C0, C2.5	21	C0, C2.5	23
	C0, C4	23	C0, C4	24	C0, C2.5	32	C0, C3	*20*	C0, C3	21	C0, C2	27
	C0, C2.5	24	C0, C2.5	25	C0, C4	37	C0, C4	25	C0, C4	24	C0, C3	30
	C6	24	C6	23	C3	33	C4	23	C4	22	C2.5	28
	C0	37	C0	37	C0	45	C0	40	C0	38	C0	51
**PO**	C1, C3, C4, C12	*14*	C1, C3, C4, C8	*15*	C1, C3, C4, C8	*16*	C1.5, C3, C4, C12	*13*	C1.5, C3, C4, C12	*13*	C1.5, C3, C4, C8	*14*
	C0, C1, C3, C4	27	C0, C1, C3, C4	32	C0, C1, C3, C4	*20*	C0, C1, C2, C4	23	C0, C1.5, C3, C4	25	C0, C1.5, C2, C4	*16*
	C0, C1, C2, C4	28	C0, C1, C2, C4	33	C0, C1, C2, C4	22	C0, C1.5, C3, C4	24	C0, C1, C2, C4	25	C0, C1.5, C3, C4	*16*
	C0, C0.5, C3, C4	32	C0, C0.5, C3, C4	35	C0, C1.5, C2, C4	24	C0, C1, C3, C4	25	C0, C1, C3, C4	26	C0, C1, C3, C4	*18*
	C1, C3, C4	21	C1, C3, C4	22	C1, C3, C4	20	C2, C4, C12	*16*	C1, C3, C8	*17*	C0.5, C3, C4	*18*
	C0, C1, C4	35	C0, C1, C4	39	C0, C1, C4	30	C0, C1, C4	30	C0, C1, C4	30	C0, C2, C4	24
	C0, C1.5, C4	38	C0, C0.5, C4	41	C0, C1.5, C4	33	C0, C2, C4	30	C0, C2, C4	33	C0, C1, C4	24
	C0, C2, C4	40	C0, C1.5, C4	42	C0, C1, C3	35	C0, C2, C3	35	C0, C1.5, C4	36	C0, C1, C3	28
	C1, C4	27	C1.5, C4	31	C1, C4	29	C1, C4	24	C1, C4	26	C1, C4	25
	C0, C2	61	C0, C2	64	C0, C4	50	C0, C3	45	C0, C3	46	C0, C3	41
	C0, C1	65	C0, C4	68	C0, C3	51	C0, C1	47	C0, C1	49	C0, C4	43
	C0, C4	66	C0, C1	69	C0, C1.5	61	C0, C2	54	C0, C2	55	C0, C2	44
	C3	45	C3	49	C4	41	C3	40	C3	40	C3	33
	C0	99	C0	99	C0	100	C0	86	C0	85	C0	86

Model 1: significant covariates included, combined error model (final model).

Model 2: significant covariates included, proportional error model.

Model 3: significant covariates included, additive error model.

Model 4: no covariates, combined error model (selected structural model): best model for B-LSS application regarding IV profiles.

Model 5: no covariates, proportional error model.

Model 6: no covariates, additive error model: best model for B-LSS application regarding PO profiles.

C_t_: concentration at time t in hours post-dose, OFV: objective function value, 95^th^ APE%: 95^th^ percentile of the absolute relative errors.

The performance of B-LSS was evaluated using the structural, final, and several related Pop-PK models that differ from the final one in terms of error models as well as the covariates included. For each model, 28 LSS (14 for IV and 14 for PO) were selected using the above performance criteria. The results of the evaluated models are shown in Table 2. It is worth emphasizing that the final model did not give the best prediction for AUC though it has the least OFV. Associated to this model, 10 LSS (9 for IV and 1 for PO) had 95^th^ PAE% of 20% or less.

### Bayesian LSS performance

Twelve B-LSS (8 for IV and 4 for PO, using models no. 4 and 6, respectively) that required 4 or less concentration-time points obtained within 4 hours post-dose, estimate AUC with 95^th^ PAE% of 20% or less, Table 2. Among these LSS, (C_0_, C_2_, C_3_, C_4_) for IV and (C_0_, C_1.5_, C_2_, C_4_) for PO CsA, had the best performance, with 95^th^ PAE% of 14% and 16%, respectively. However, it is possible to reduce the prediction error if a prolonged sampling period beyond 4 hours post-dose is allowed. For example, the LSS (C_2_, C_2.5_, C_8_, C_10_) had a reduced PAE% of 7% for IV CsA.

Furthermore, the prediction of the ‘underlying’ AUC revealed that the selected B-LSS often had a better performance when the ‘underlying’ AUC was estimated rather than the observed AUC. Indeed, under the same conditions of 4 or less sampling points within 4 hours post-dose, we identified 15 B-LSS (instead of 12), that have 95^th^ PAE% of 20% or less, Table 3.

**Table 3:** Performance of B-LSS for the prediction of observed and ‘underlying’ AUC using selected Pop-PK models

	**LSS #**	**Concentration-time points**	**Error indices (for observed AUC)**						**Error index for ‘underlying’ AUC**
			**RMSE% (Confidence interval)**	**ME% (Confidence interval)**	**E%**			**95**^**th **^**PAE%:**	**95**^**th **^**PAE%:**
					**<-20%**	**[-20%, 20%]**	**>20%**		
**IV**	1	C2, C2.5, C8, C10	4.39(3.26, 5.28)	1.19(-0.67, 3.06)	0	23	0	*7*	*14*
	2	C0, C2, C3, C4	7.30(4.37, 9.36)	1.93(-1.18, 5.04)	0	23	0	*14*	*17*
	3	C0, C2, C2.5, C3	7.71(4.32, 10.01)	1.75(-1.57, 5.07)	0	22	1	*15*	*17*
	4	C0, C2, C2.5, C4	7.61(2.33, 10.51)	2.83(-0.30, 5.95)	0	22	1	*16*	*19*
	5	C2, C2.5, C8	5.70(4.30, 6.81)	3.24(1.17, 5.31)	0	23	0	*10*	*17*
	6	C0, C2, C3	8.14(4.63, 10.54)	2.40(-1.04, 5.84)	0	22	1	*16*	*19*
	7	C0, C2.5, C3	9.85(7.16, 11.95)	-0.61(-4.96, 3.73)	0	23	0	*18*	*14*
	8	C0, C2.5, C4	9.53(6.42, 11.85)	0.89(-3.30, 5.09)	0	23	0	*19*	*16*
	9	C2.5, C8	8.64(6.49, 10.36)	0.58(-3.24, 4.39)	0	23	0	*15*	*14*
	10	C0, C2.5	11.33(8.25, 13.74)	1.05(-3.93, 6.04)	1	22	0	*20*	*19*
	11	C0, C3	11.42(8.55, 13.69)	-0.94(-5.97, 4.09)	1	22	0	*20*	*15*
	12	C0, C4	12.11(8.19, 15.04)	2.65(-2.58, 7.87)	1	21	1	25	*19*
	13	C4	13.88(10.30, 16.71)	4.36(-1.46, 10.19)	1	18	4	23	25
	14	C0	23.32(16.81, 28.37)	0.55(-9.75, 10.86)	6	10	7	40	38
**PO**	15	C1.5, C3, C4, C8	6.98(5.29, 8.33)	-3.20(-5.24, -1.17)	0	39	0	*14*	*16*
	16	C0, C1.5, C2, C4	7.50(4.97, 9.36)	0.70(-1.76, 3.15)	0	38	1	*16*	*11*
	17	C0, C1.5, C3, C4	7.60(5.48, 9.25)	-0.35(-2.85, 2.14)	0	39	0	*16*	*14*
	18	C0, C1, C3, C4	9.34(6.62, 11.42)	0.43(-2.63, 3.50)	1	38	0	*18*	*16*
	19	C0.5, C3, C4	11.73(7.27, 14.91)	0.08(-3.77, 3.93)	1	38	0	*18*	*15*
	20	C0, C2, C4	11.02(8.00, 13.37)	3.40(-0.04, 6.84)	1	36	2	24	*19*
	21	C0, C1, C4	10.26(6.11, 13.15)	0.48(-2.89, 3.84)	1	37	1	24	*20*
	22	C0, C1, C3	13.08(9.20, 16.04)	2.07(-2.17, 6.31)	2	34	3	28	21
	23	C1, C4	11.96(8.36, 14.70)	-2.86(-6.68, 0.95)	4	34	1	25	25
	24	C0, C3	18.65(11.05,23.94)	2.16(-3.92, 8.24)	4	31	4	41	34
	25	C0, C4	21.37(16.26,25.47)	2.09(-4.89, 9.07)	6	23	10	43	38
	26	C0, C2	21.19(12.55,27.21)	8.61(2.25, 14.97)	2	29	8	44	36
	27	C3	17.46(12.77,21.14)	-3.17(-8.81, 2.47)	7	29	3	33	30
	28	C0	43.26(31.81,52.26)	15.62(2.37, 28.87)	7	14	18	86	87

The selected Pop-PK models are the structural model with combined errors (Model 4 in Table 2) and the one with additive errors (Model 6 in Table 2) for IV and PO CsA, respectively.

C_t_: concentration at time t in hours post-dose, ME%: relative mean prediction error, RMSE%: relative root mean squared prediction error, 95^th^ APE%: 95^th^ percentile of the absolute relative errors.

## Discussion

Limited sampling strategies are gaining ground over extensive sampling in the drug development process and clinical practice, particularly in pediatric therapies. With the increasing use of Pop-PK modeling and Bayesian philosophy in drug R&D, we can notice the recent transition from classical regression LSS approach towards B-LSS. The current work investigates the use of B-LSS in the estimation of AUC, for CsA administered through IV or PO routes in pediatric hematopoietic stem cell transplantation. Taking into account clinical considerations, our approach uses the empirical Bayesian method as implemented in NONMEM^®^ for the selection of the smallest set of sampling points (i.e. LSS) that allow accurate estimation of individual AUC.

Through LSS development process, we have been led to question the appropriateness, for B-LSS application, of the final Pop-PK model, particularly when its development is mainly driven by the objective function value OFV. For this, we have tested several related Pop-PK models and found that the usually referred as the final one does not necessarily provide the best B-LSS performance for AUC prediction. This is in fact not counterintuitive since this final model is chosen under curve fitting criteria. The PK parameters found through this goodness of fit criteria might not give the best estimation of AUC, which is indeed a summary of the information carried by the concentration curve. It would be interesting in the future to directly integrate an additional constraint that minimizes prediction errors in AUC, within the model optimization process, in order to account for both curve fitting and AUC estimation.

The prediction error of B-LSS depends on the Pop-PK-model used to predict drug concentrations. Several Pop-PK model components such as covariate and error models can significantly influence the performance of B-LSS. In a standard Pop-PK model development, reduction in model objective function value OFV is the main criterion to judge the quality of the model. In this approach, Pop-PK parameters are estimated and optimized via a restricted *maximum likelihood* method implemented in NONMEM^®^. However, for B-LSS application to estimate AUC, a more efficient selection of Pop-PK models can be achieved by the additional consideration of the impact of Pop-PK model components on AUC prediction rather than only considering their impact on PK parameters estimation. In our case, for example, even though the structural model with combined error shows a better overall fit for PO profiles, it underestimates C_max_ of individual profiles. The structural model with additive error allowed a better estimation of C_max_ that has the main contribution to AUC value calculated by trapezoidal method and therefore this model is associated with a better performance of B-LSS.

To develop B-LSS for CsA in pediatric hematopoietic stem cell transplantation and investigate its performance, we developed a Pop-PK model following the standard procedure, from the structural model to the final covariate model, while carefully keeping the intermediate tested models for comparison. In order to identify the model that best predicts AUC, the performance of the final model was compared with intermediate ones that differ in one or more model components. For each model, all possible one, two, three, or four concentration-time point LSS were investigated and their predictive performance evaluated. Moreover, we studied the situation when B-LSS are targeting the ‘underlying’ AUC rather than the observed AUC and found that the B-LSS prediction performance is improved. Indeed, we have used the two models (no. 4 and 6 in Table 2 for IV and PO CsA, respectively), which have the smallest prediction errors for the observed AUC, and obtained a better performance when the ‘underlying’ AUC was estimated.

The studied population covers a wide range of demographic and clinical characteristics that enables large applicability of the developed LSS. In addition, to avoid the overestimation of the predictive performance, the data set used for validation has to be different from the one used for learning. However, the small number of initially available PK profiles, a common issue in pediatric research, led us to use leave-one-out-cross-validation approach. This method is generally used as an alternative to compensate for small data sets. When evaluating the LSS performance, relative errors indices, namely E%, ME% and RMSE%, were computed. However, we are aware that the use of relative errors might induce the bias when applied to highly asymmetrical data, thus their distribution was considered [28]. The 95^th^ PAE% was used to initially compare B-LSS performance for the Pop-PK models since it is more frequently used in clinical setting for the evaluation of errors. Other error indices are calculated as well for all considered models and the detailed results are reported in Table 3 for the best performing ones, namely, for models no. 4 and 6 of Table 2. Particularly, the 12 proposed B-LSS (8 IV and 4 for PO CsA) were verified for the absence of bias and their ME% were not significantly different from 0. Even though the LSS developed in this study allow accurate and precise CsA AUC estimation, we have to mention that further prospective trials are still needed to determine whether AUC-based monitoring can increase efficacy and avoid toxicity. However, evaluating the value of AUC as a marker for therapeutic drug mentoring is outside the scope of this paper.

In the current study, using standard model diagnostic criteria, we have constructed a two compartment Pop-PK model with lag time and combined error to characterize CsA PK data, which is in agreement with previous hematopoietic stem cell transplantation studies [11,13,17,20,29]. In our structural model, CL was estimated to be 14.8 L/h with an IIV of 31% (14.8, 31%), which are similar to the reported ones for pediatric patients (11.3, 36%) [17] and (15.3, 17%) [20]. However, higher values of CL were reported for adult populations (22.3, 27.7%) [11], (25.4, 38.7%) [13], and (52, 42%) [29]. The covariate influence on CL was described in two studies. Willemze et al. [7] has shown power and linear relationships between CL and WT as well as CL and time post transplantation (TPT), respectively. Kim et al. [29] reported linear relationships between CL and sex as well as hematocrit. In our study, WT was included as an allometric covariate for both CL and V_C_, which is in agreement with the findings of Willemze et al. [7] and generally adopted in pediatric PK modeling [24]. In addition, we found relationships between CL and alkaline phosphatase (AP) as well as age at profile date (AG), the former relationship is compatible with the fact that CsA has hepatic metabolism and that its elimination depends on liver function. Hematopoietic stem cell transplantation complication includes chronic and acute liver graft-versus-host disease for which alkaline phosphatase is a clinical marker [30,31]. In addition our investigation confirmed the inverse correlation between age and two PK parameters, namely, CL and Vc [15,16]. Moreover, our results regarding the central and peripheral volume of distribution were within the range of the reported studies [11,13,17,20,29], where values largely vary (Vc: 12.9-52, Vp: 59.9-496). Furthermore, a lag time for CsA absorption was previously reported in three studies [11,13,17]. The present investigation showed the influence of two clinically relevant covariates on lag time, namely, time post transplantation and FORM. In hematopoietic stem cell transplantation patients, time post transplantation is related to the intestinal integrity that can affect CsA absorption [9] and, as expected, capsules need additional time to be available for absorption when compared to suspension. KA value was higher than that reported in adults [11,13,29] and close to estimates of Willemze et al. in pediatrics [17]. The CsA bioavailability in our study was estimated to be 59% with an IIV of 30%, which compares well with reported values [11,17].

## Conclusion

B-LSS requiring 4 or less concentration-time points obtained within 4 hours post-dose can estimate CsA AUC in pediatric hematopoietic stem cell transplantation with acceptable prediction errors. However, the Pop-PK model developed using the standard model diagnostic criteria, does not always lead to the best model for B-LSS application. As we have seen in this paper, even the final covariate model gives a better fitting for concentration data in the sense of objective function value (OFV) than the structural model, the latter has a better AUC prediction than the former. Thus, for improved B-LSS application, more considerations with focus on the error in AUC prediction have to be taken into account in the development of Pop-PK models. Moreover, in the case where the prediction of the ‘underlying’ AUC is preferred compared to the observed AUC, as the residual error is excluded in the former, B-LSS can have a better performance.

## Appendix

### Pop-PK model development

Population pharmacokinetic analyses were performed using the nonlinear mixed effect model approach as implemented in NONMEM^®^ software (Version VII). The first order conditional estimation with interaction (FOCE-I) method was used to determine PK parameters.

To define the structural model, one, two and three compartment models with first-order absorption and elimination were used to analyze available CsA data. The lag time in absorption was also tested for each model. The exponential model was used to describe inter-individual variability for PK parameters as expressed in Eq.7:

(7) $$\theta_{\mathrm{ij}}=\theta_{j}\times \;\text{EXP}\left(\eta_{\mathrm{ij}}\right)$$

where θ_ij_ is the j^th^ PK parameter for the i^th^ individual, θ_j_ is the typical value of the population parameter; ɳ_ij_ is a random variable characterizing the between subject variability. A combined version of additive and proportional models was used to test for the residual variability (Eq.8):

(8) $$C_{\text{obs}}=\;C_{\text{pred}}\times \;\left(1+\epsilon_{1}\right)\;+\epsilon_{2}$$

where C_obs_ and C_pred_ are the observed and predicted CsA blood concentrations, respectively; ε_1_ and ε_2_ are random variables describing the unexplained residual variability.

The structural model was developed based on statistical significance in the reduction of the objective function value (OFV) using likelihood ratio (LT) test, as well as other criteria such as the model stability and the improvement in model fitting. As usually done in pediatric Pop-PK modeling, weight was initially integrated as an allometric scaling factor for the clearance and volume of distribution. The covariate model was established using the forward inclusion backward elimination method. This approach was accomplished through three steps. In the first step, we set up the basic model by including weight as an allometric scaling factor for the clearance and volume of distribution into the structural model. Scatter plots of model parameters against each covariate helped to evaluate the potential covariate impact and the relation patterns. In the second step, each candidate covariate was screened in turn by incorporating it into the basic model to develop the intermediate models toward a full one. The difference in OFV obtained for a model with n + 1 covariates and the nested one with n covariates approximates the χ^2^ distribution with one degree of freedom, and a change of OFV greater than 6.63, associated with a p-value of 0.01, was considered for statistical significance. The following covariate were considered: weight (WT), age at profile date (AG), time post transplantation (TPT), sex, dosage form (FORM), co-administration of corticosteroid, calcium channel blacker and azole antifungal, blood urea nitrogen, albumin, total protein, total bilirubin, aspartate aminotransferase (ALT), alanine aminotransferase (AST), gamma glutamyl transpeptidase (GGP), alkaline phosphatase (AP), hemoglobin, hematocrit , and red blood cell count. Only potentially clinically meaningful relationships were considered. Hence, we have tested the influence on CL of WT, AG, sex, FORM, co-administration of corticosteroid, calcium channel blacker and azole antifungal, blood urea nitrogen, albumin, total protein, total bilirubin, ALT, AST, GGP, AP, hemoglobin, hematocrit and red blood cell count; we have tested the influence on Vc, Q, and Vp of WT, AG, and sex; and finally tested the influence on KA and ALAG1 of AG, sex, TPT, and FORM. Sex, FORM, and co-medications were included in the model as categorical covariates in a linear mode. Other covariates were included as continuous ones in linear, exponential, and power modes; these covariates are centered to their median values. In the backward step, each covariate was independently removed from the full model to confirm its importance. An increase in OFV of more than 7.87 (p-value, 0.005) was required to confirm that the covariate was significant. The final Pop-PK model included all significant covariates. The Perl speaks NONMEM (PsN) toolkit was used for stepwise covariate model building [25].

### Pop-PK results

The initial analyses without covariates showed that a two-compartment model with lag time and combined error model described the CsA PK profile better than the other tested models. Thus, this is chosen as the structural model in the current study. The estimated PK parameters were CL, Vc, Vp, Q, KA, ALAG, and F. Inter-individual variability OMEGA can be estimated for CL, Vc, Q, KA and F; inter-individual variability of Vc is highly correlated with that of CL and was estimated as a linear function of it. Parameter estimates for the structural model with combined and additive error model are shown in Table 4.

**Table 4:** Parameter estimates for the two structural Pop-PK models selected for B-LSS application

		**Structural Pop-PK model with combined residual error**				**Structural Pop-PK model with additive residual error**			
**PK parameters**		**NONMEM fixed effect parameters**		**Inter-individual variability%**		**NONMEM fixed effect parameters**		**Inter-individual variability%**	
		**estimate**	**RSE%**	**estimate**	**RSE%**	**estimate**	**RSE%**	**estimate**	**RSE%**
**CL**		14.82	7	31	14	14.49	8	32	15
**Vc**		31.8	9	†		24.91	10	†	
**Q**		13.49	13	80	11	13.14	15	100	14
**Vp**		104.6	10	-	-	86.15	8	-	-
**KA**		0.71	16	83	11	0.58	13	75	10
**ALAG**		0.39	6	-	-	0.39	6	-	-
**F**		0.61	10	32	24	0.61	11	29	24
**θ8**		0.86	10	-	-	1.02	15	-	-
**Cov (CL, Q)**		-	-	44	27	-	-	48	22
**Residual error**	Prop.	17.5				-			
	Add.	15 ng/mL				100 ng/mL			

The selected Pop-PK models are the structural model with combined errors (Model 4 in Table 2) and the one with additive errors (Model 6 in Table 2) for IV and PO CsA, respectively.

^†^Inter-individual variability (Vc) = Inter-individual variability (CL) × θ8.

CL: clearance, Vc: apparent volume of distribution of the central compartment, Vp: apparent volume of distribution of the peripheral compartment, Q: inter-compartmental transfer rate, KA absorption rate, ALAG: lag time in oral absorption, F: oral bioavailability, RSE%: relative standard errors.

**Figure 4:**
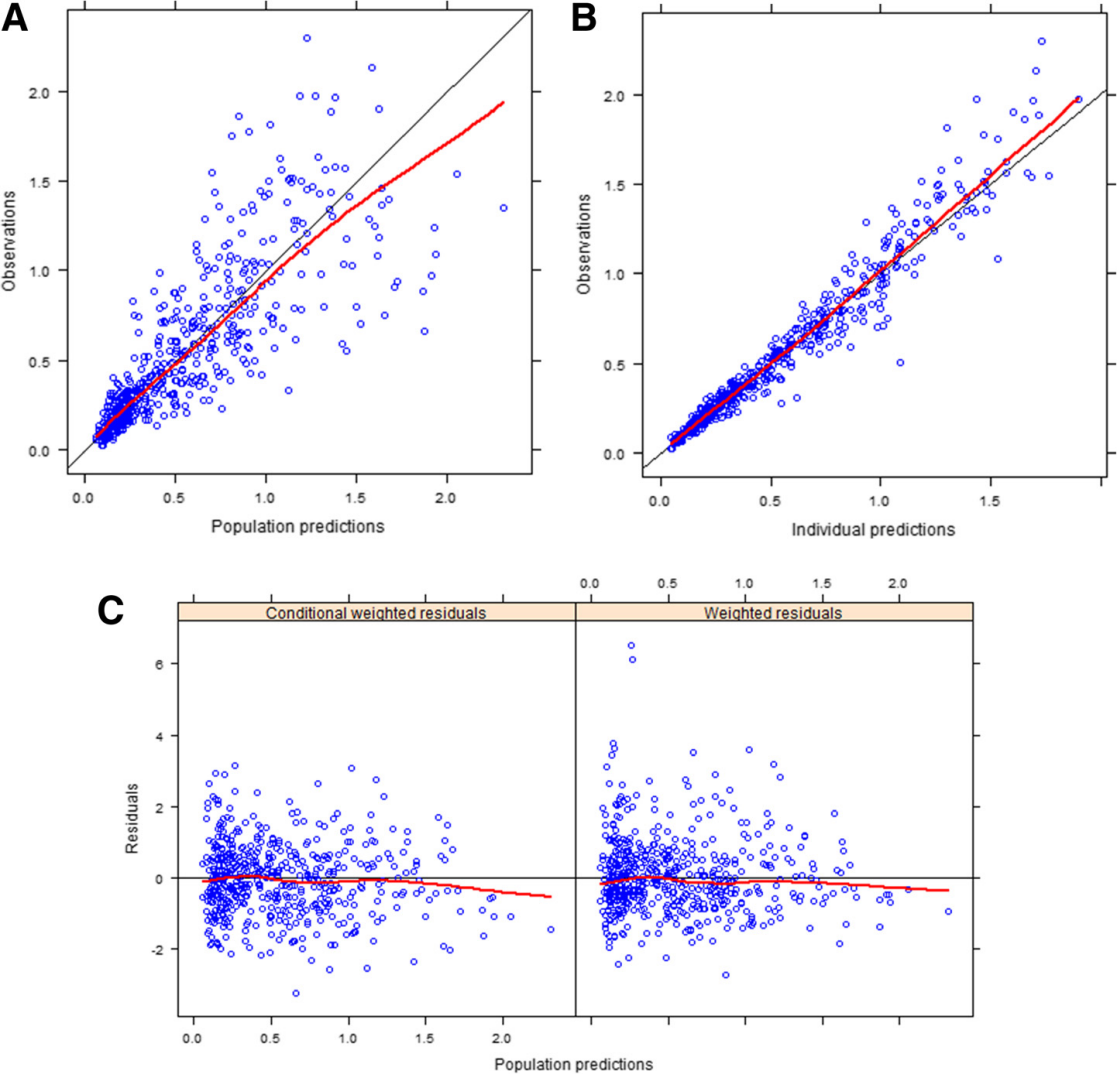
**Graphical model evaluation. A**: relationship between the observed and the predicted CsA concentrations based on the final parameter estimates (PRED); **B**: relationship between the observed and the individual predicted concentrations (IPRED); **C**: distribution of weighted residual (WRES) and conditional weighted residuals (CWRES).

The final model comprises the following covariates: WT, AG, TPT, and AP, and FORM. The estimated parameters are reported in Table 5. The relative standard errors (% RSE) of the parameters were acceptable, with a range from 0.05 to 0.30. Figure 4A shows the relationship between the observed and the predicted CsA concentrations based on the final parameter estimates (PRED). Figure 4B shows the relationship between the observed and the individual predicted concentrations (IPRED). Both plots show good correlation, suggesting that the final model explains well the observed data, although peak concentrations in several individuals were slightly underestimated by PRED. The values and distribution of weighted residual (WRES) were unsatisfactory confirming the adequate use of FOCE-I instead of FO as an estimation method. The conditional weighted residuals (CWRES) for model-predicted concentrations shown in the narrow rectangular distribution in function of observed concentration and time, Figure 4C, are also acceptable.

**Table 5:** Final Pop-PK model parameter estimates

**PK parameters**		**NONMEM fixed effect parameters**		**Inter-individual variability%**	
		**Estimate**	**RSE%**	**Estimate**	**RSE%**
**CL**		θ1 = 15.66	5	17	11
		θ10 = -0.32	19		
		θ11 = 0.0017	29		
**Vc**		θ2 = 36.68	9	2	7
		θ13 = -0.39	16		
**Q**		θ3 = 14.71	9	55	15
		θ12 = 0.023	18		
**Vp**		θ4 = 105	10	-	-
**KA**		θ5 = 0.8	15	72	10
**ALAG**		θ6 = 0.46	3	-	-
		θ8 = 0.005	5		
		θ9 = -0.39 for suspension	19		
		θ9 = -0.022 for capsule	18		
		θ14 = -0.014	30		
**F**		θ7 = 0.59	8	30	15
**Residual error**	Prop.	16			
	Add.	19 ng/mL			

CL = THETA (1) × ((WT/36.1) **0.75) × ((AG/11.82) **THETA(10)) × (1 + THETA(11) × (AP-99)) × EXP(ETA(1)).

Vc = THETA(2) × (WT/36.1) × ((AG/11.82) * * THETA(13)) × EXP(ETA(2)).

Q = THETA(3) × (1 + THETA(12) × (WT - 36.1)) × EXP(ETA(3)).

Vp = THETA(4).

KA = THETA(5) × EXP(ETA(4)).

ALAG = THETA(6) × EXP(THETA(8) × (TPT - 3.71)) × (1 + THETA(9)) × (1 + THETA(14) × (AG- 11.82)).

F = THETA(7) × EXP(ETA(5)).

CL: clearance, Vc: apparent volume of distribution of the central compartment, Vp: apparent volume of distribution of the peripheral compartment, Q: inter-compartmental transfer rate, KA absorption rate, ALAG: lag time in oral absorption, F: oral bioavailability, WT: weight, AG: age at profile date, TPT: time post transplantation, AP: alkaline phosphatase, FORM: dosage form, RSE%: relative standard errors.

## Abbreviations

95^th^ PAE%: 95^th^ percentile of absolute values of relative prediction errors; AUC: Area under the concentration-time curve; Observed AUC: AUC estimated using the trapezoidal method and observed concentrations; Predicted AUC: AUC estimated using LSS method and observed concentrations; Underlying AUC: AUC estimated using the trapezoidal method and individual predicted concentrations without residual error; B-LSS: Bayesian limited sampling strategy; CsA: Cyclosporine; C_t_: Concentration at time t in hours post-dose; E%: Relative error; IV: Intravenous administration; LSS: Limited sampling strategy; ME%: Mean relative prediction error; OFV: Objective function value; PO: Oral administration; Pop-PK: Population pharmacokinetic; RMSE%: Root mean squared relative prediction error.

## Competing interests

The authors declare that they have no competing interests.

## Authors’ contributions

SS participated in study design, performed data analysis and interpretation, and drafted the manuscript. JL, FN, participated in study design and coordination, data analysis and interpretation, and helped to draft the manuscript. OB helped to perform data analysis. CL, YT and AL conceived the clinical study, and participated in its design and coordination, and helped for acquisition of data. All authors read and approved the final manuscript.
